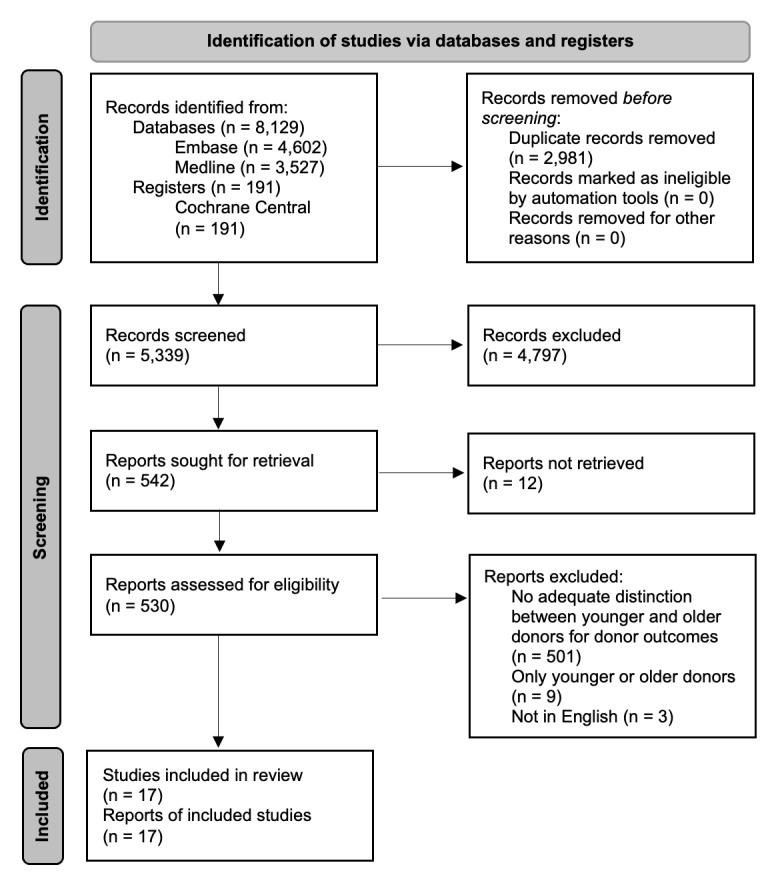# Older living liver donors can enlarge the donor pool: a systematic review and meta-analysis

**DOI:** 10.1097/JS9.0000000000001419

**Published:** 2024-04-03

**Authors:** Hayo W. ter Burg, Alicia J. Chorley RN, Wojciech G. Polak, Leonieke W. Kranenburg, Markus U. Boehnert, Robert C. Minnee

**Affiliations:** a Erasmus MC Transplant Institute, University Medical Center Rotterdam, Department of Hepato-Pancreato-Biliary (HPB)/Transplant Surgery; b University Medical Center Rotterdam, Department of Psychiatry, Rotterdam, The Netherlands; c King Faisal Specialist Hospital and Research Center, Organ Transplant Center of Excellence, Riyadh, Saudi Arabia

**Keywords:** complications, liver transplantation, living donors, mortality, quality of life

## Abstract

**Background:**

Living donor liver transplantation (LDLT) is the best option for end-stage liver disease patients. Older potential donors are increasingly requesting donation. This study aims to systematically assess the differences in donor perioperative and postoperative complications, mortality, and quality of life (QoL) between younger and older living liver donors.

**Materials and methods:**

Embase, Medline, and Cochrane were searched for studies published between 2002 and 2 June 2023. Donor complications, major complications, biliary complications, mortality, and QoL were systematically reviewed, including meta-analyses. Donors aged >50 years were considered older. The methodological quality of the studies was assessed using the Newcastle–Ottawa quality assessment Scale.

**Results:**

The search yielded 8320 studies, of which 17 were included. The risk ratio (RR) for complications in younger donors was 1.08 [0.90–1.31] (*P*=0.41). RRs for major complications in younger donors were 0.98 [0.64, 1.48] and 0.89 [0.50, 1.57] using Clavien–Dindo ≥III and ≥IIIb as major complication. RR for biliary complications in younger donors was 1.59 [1.05–2.42] (*P*=0.03). Mortality rate in younger donors was 47/13 238 (0.4%) and in older donors 13/989 (1.3%). Physical component summary (PCS) in younger donors was 51.87 and in older donors 51.29. Mental component summary (MCS) in younger donors was 52.93 and in older donors 55.40.

**Conclusion:**

Older donors do not have a higher complication or mortality rate than younger donors after LDLT. They may have a lower rate of biliary complications. Additionally, older donors have a similar QoL after LDLT. With careful selection, older donors can be included in screening programs for living liver donation to expand the donor pool.

## Graphical abstract

**Figure:**
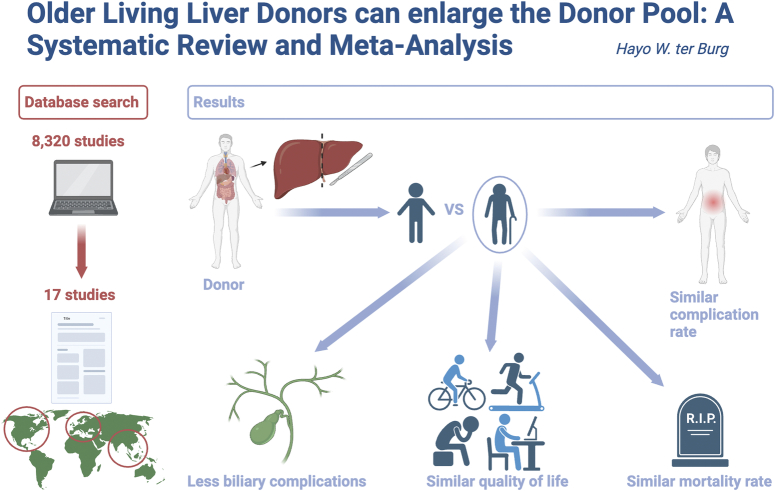

Highlights

- Older living liver donors do not have a higher complication rate than younger donors.
- Neither do they have a higher mortality rate.
- Older living liver donors have a similar quality of life after donation.
- Older donors can be included in screening programs to expand the donor pool.

Living donor liver transplantation (LDLT) is a relatively recent medical procedure in addition to the existing deceased donor liver transplantation. However, surgical expertise has developed quickly, and nowadays, LDLT is considered the best option for patients with end-stage liver disease with respect to graft and patient survival^1^. It provides the best outcomes due to reduced waiting times, shorter cold ischemia times, and allowing planned pre-emptive transplantation^2^.

During the screening process for LDLT, living liver donors undergo evaluation based on multiple criteria. Key criteria are remnant liver volume, hepatic steatosis, and donor age, each with cut-off values^3^. A recent systematic review and meta-analysis concluded that a remnant liver volume of less than 30% should not be accepted^4^. The maximal hepatic steatosis accepted depends on the remnant liver volume^3,5^. A commonly used maximum donor age is 50 years. Furthermore, the population is aging worldwide and as a result, more and more potential donors above these ages are requesting donation. Meanwhile, little is known in the literature about the impact of living liver donation on older donors^6^. In living kidney donation, the eligibility criteria have already shifted to older donors, providing safe and comparable results compared to younger donors^7,8^. If donor safety can be guaranteed, shifting these criteria to older donors for living liver donation is necessary as well. To guarantee donor safety, this study aims to systematically assess the differences in donor perioperative and postoperative complications, mortality, and quality of life (QoL) between younger and older living liver donors. The results of this study may contribute to expanding the eligibility criteria for living liver donors and therefore enlarging the donor pool.

## Materials and methods

This study was reported in line with PRISMA (Preferred Reporting Items for Systematic Reviews and Meta-Analyses) and AMSTAR (Assessing the Methodological Quality of Systematic reviews) Guidelines^9,10^. The protocol was prospectively registered on the International Prospective Register of Systematic Reviews (PROSPERO).

### Outcomes

The primary outcome was differences in donor perioperative and postoperative complications between living liver donors aged ≤50 and >50 years. The secondary outcome was differences in donor perioperative and postoperative mortality between living liver donors aged ≤50 and >50 years. The tertiary outcome was differences in donor postoperative QoL between living liver donors aged ≤50 and >50 years, measured using the Short Form-36 Health Survey (SF-36). We focused on the physical (PCS) and mental component summary (MCS) scores.

### Search strategy

A search strategy was created using the key terms ‘living donor liver transplant’, ‘living donor liver transplantation’, ‘LDLT’, ‘mortality’, ‘survival’, ‘quality of life’, ‘QoL’, ‘complication’, and ‘reoperation’. A literature search was performed using Embase, Medline ALL, and Cochrane Central Register of Controlled Trials on 2 June 2023. The full search strategy can be found in SDC, Appendix 1, Supplemental Digital Content 1, http://links.lww.com/JS9/C321. Furthermore, a snowball search was performed by manually searching the reference lists of included studies.

### Inclusion and exclusion criteria

Inclusion criteria were case series, cohort studies, case–control studies, research population of living liver donors with subgroups for age ≤50 and >50 years, written in English, published between 2002 and 2 June 2023.

Exclusion criteria were (systematic) reviews, meta-analyses, case reports, editorials, animal, and nonhuman studies, and letters to the editor.

### Study selection

According to the inclusion and exclusion criteria, two reviewers independently and blinded to each other’s decisions reviewed all studies based on title and abstract with the help of RefWorks (ProQuest) reference management software. Subsequently, they screened the studies on full text. Disagreements about study selection were resolved by discussion with a third reviewer. Microsoft Word was used for recording decisions.

### Data extraction

From the study documents the following data was extracted: study ID (i.e. first author, year of publication), country, sample size, donor age, donor-recipient type, surgery techniques, date of surgery, follow-up, data collection period, data collection point, donor complications, donor mortality, and donor QoL.

One reviewer extracted the data, and a second reviewer checked the extracted data. Disagreements were resolved by discussion with a third reviewer.

When data was missing, first, study investigators were contacted by e-mail to obtain this data. In addition, they were asked for unreported data or additional details. When the missing data could not be obtained, the data as stated in the study was used.

The data was recorded in Microsoft Excel and Cochrane Review Manager 7.4.0. For data extraction and management Cochrane Review Manager 7.4.0 was used.

### Quality assessment

Two reviewers independently assessed the methodological quality of the studies using the Newcastle–Ottawa quality assessment Scale (NOS)^11^. Disagreements were resolved by discussion with a third reviewer.

Characteristics assessed: the selection of the study groups, the comparability of the groups, and the ascertainment of either the exposure or outcome of interest for case–control or cohort studies, respectively^11^. The assessment was done at study level.

Studies with poor quality according to the NOS were excluded from the data synthesis.

### Data analysis

A minimum of three studies on the outcome was needed to conduct a meta-analysis.

For donor complications and mortality, the results of the included studies were systematically described, and a table of evidence was made showing study ID, method (i.e. country, donor-recipient type, surgery techniques, date of surgery), sample size, donor age, follow-up, complications, and mortality. Meta-analyses were conducted on the studies using Cochrane Review Manager 7.4.0. Forest plots for differences in complications between younger and older donors were made. The Mantel–Haenszel statistical method for dichotomous data was used. Fixed effects were used for the analysis model, as we expected the studies to differ little from one another. As effect measure the risk ratio was used. The weights, risk ratios, and 95% CI were presented. *I*² identified statistical heterogeneity between studies. *I*²≥50% indicated high heterogeneity. *P*<0.05 was considered statistically significant. Sensitivity analyses (to eventually explore the source of heterogeneity) were done for the effect measures odds ratio and risk difference, and for the random effects analysis model. Funnel plots were made to check for the existence of nonreporting bias. Subgroup analyses were performed for major donor complications and donor biliary complications.

For donor QoL, the results of the studies were systematically described, and a table of evidence was made showing study ID, method (i.e. country, donor-recipient type, surgery techniques, data collection period, and data collection point), sample size, donor age, and donor QoL.

## Results

### Search result

**Figure 1:**
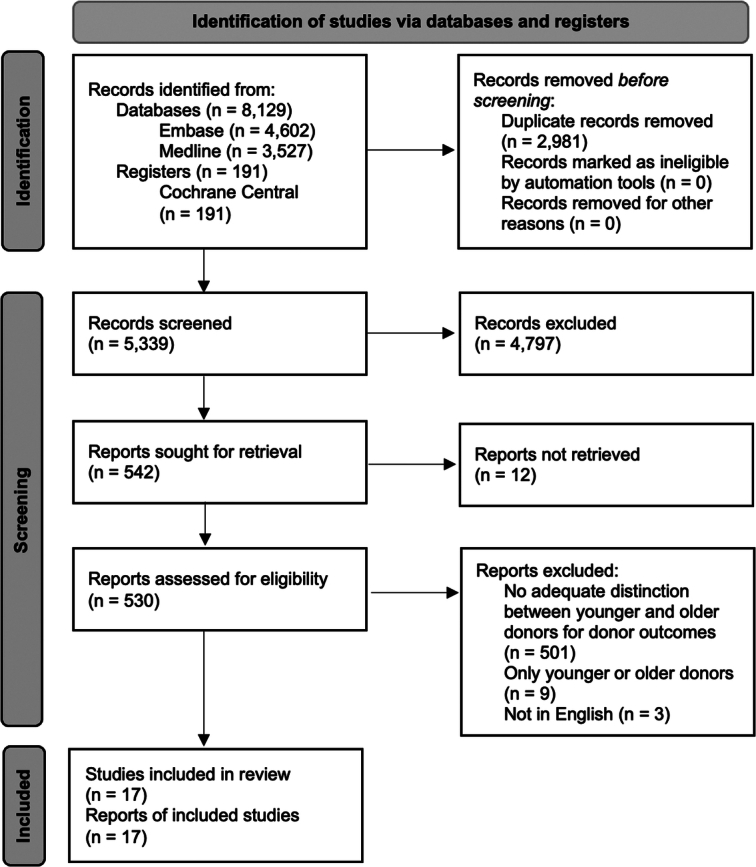
PRISMA 2020 flow diagram for new systematic reviews which included searches of databases and registers only.

Our literature search yielded a total of 8320 potentially eligible studies. Five hundred and thirty studies were assessed for eligibility. After reading the full text, 501 studies were excluded because they made no adequate distinction between younger and older donors for donor outcomes, nine because they contained only younger or older donors, and three because they were not in English. Seventeen studies^12–28^ met our inclusion and exclusion criteria and were included. Of those, 15^12–26^ discussed donor complications and mortality, and 2^27,28^ QoL. The PRISMA flow diagram of the study selection is shown in Figure 1.

### Complications and mortality

#### Characteristics of the included studies

The characteristics of the included studies are shown in Table 1. Ten studies were from Asia^13–22^, three from North America^12,23,24^, and two from Europe^25,26^. These studies discussed a total of 17 813 donors. The right lobe was donated in 10 169 cases (57.1%), the left lobe in 1272 cases (7.1%), the left lateral segment in 1255 cases (7%), the extended right lobe in 34 cases (0.2%), and the right posterior segment in 12 cases (0.1%). In 5071 (28.5%) cases, the surgery technique was missing or unclear.

**Table 1:** Characteristics of the 15 included studies on donor complications and mortality.

Author, year	Country	Sample size (≤50 vs >50)	Donor age	Donor-recipient type	Surgery technique	Date of surgery	Results
Akamatsu, 2007^14^	Japan	299 (237 vs 62)	Range=20–65 years	3^rd^ degree consanguinity or spouse Deviation from this was discussed by transplant team and institutional ethics board	RL (155), LL (125), LLS (19)	Jan. 1996–July 2006	Complications: postoperative complications <50=20/237 (8.4%) ≥50=4/62 (6.5%) 16 bile leakages (13 in group Y, 3 in group O), 3 gastroduodenal ulcers (group Y), 1 postoperative bleeding (group Y), and 5 gastric stases (4 in group Y, 1 in group O). No significant differences between the groups. Mortality No significant differences in survival for donors between groups. Follow-up Median of 3.7 years (range=0.3–10.8)
Dayangac, 2011^25^	Turkey	150 (122 vs 28)	Range=18–63 years	4^th^ degree consanguinity or approval from ethics committee	RL (150)	Oct. 2004–Apr. 2009	Complications <50=39/122 (28.6%) <50 vs ≥50; *P*=0.8 ≥50=8/28 (32%) Mortality There were no deaths. Follow-up Median of 3.4 years (range=1–5.6)
Goldaracena, 2016^23^	Canada	469 (378 vs 91)	Range=17–60 years	In group Y 65% blood related, in group O 50%	RL (469)	Apr. 2000–May 2014	Complications <50=48/378 (12.7%) ≥50=9/91 (9.9%) No difference in complication rate between group Y and O. In group Y, 52 complications occurred in 48 donors, in group O, 11 in 9 donors. Mortality No donor death occurred. Follow-up Median of 5.8 (range=0.1–14.3) vs 5.3 years (range=0.3–14.8) for the group Y and O, respectively (*P*=0.89)
Hong, 2021^13^	South Korea	10116 (9547 vs 569)	Range=≥16 years	3^rd^ degree consanguinity or an intense emotional relationship	RL (4701), LL (517), missing (4898)	Feb. 2000–Dec. 2015	Mortality: long-term mortality 16-29=18/5317 (0.3%) 30–39=16/2787 (0.6%) 40–49=8/1443 (0.6%) 50–59=9/519 (1.7%) 16-29 vs 50–59=6.19; *P* <0.001* ≥60=2/50 (4%) 16–29 vs ≥60=16.49; *P* <0.001* 5 deaths (9.4%) associated with liver disease: one hepatic failure immediately after donor hepatectomy, one acute hepatitis A, one liver cirrhosis, and two alcoholic liver diseases. Follow-up Median of 5.7 years (range=0–15.9)
Kadohisa, 2020^15^	Japan	470 (353 vs 117)	Range=21–70 years	3^rd^ degree consanguinity or spouses: child (117), parent (196), spouse (85), sibling (49), others (23)	RL (173), LL (156), LLS (132), RPS (9) Donors ≥65 years: LL or LLS	Dec. 1998–Mar. 2017	Complications 20–29=34/109 (31.2%) 30–39=56/157 (35.7%) 40–49=23/87 (26.4%) 50–59=19/81 (23.5%) ≥60=7/36 (19.4%)
Kim, 2012^16^	South Korea	500 (449 vs 51)	Range=18–65 years	Biologically related: parent (12), child (240), sibling (82), other (29) Not biologically related: spouse (69), other (68)	RL (500)	May 1999–Feb. 2011	Complications → only age separation for biliary complications & hyperbilirubinemia <50=79/449 (17.6%) ≥50=9/51 (17.6%) 108/500 donors (21.6%) experienced >1 complication, 139 (27.8%) complications occurred in total. C-D I, II, and III complications were 10.6%, 7.8%, and 9.4%, respectively. Mortality No donor mortality. Follow-up Median of 5 years (range=0.5–12.2)
Lauterio, 2016^26^	Italy	246 (220 vs 26)	Range=18–64 years	Biologically related: parent (25), child (138), sibling (44), other (3) Not biologically related: spouse (26), other (10)	RL (220), LL (10), LLS (16)	Mar. 2001–Dec. 2014	Complications ≤50=64/220 (29.1%) >50=12/26 (46.2%) ≥1 complication in 82 (33.3%) donors, while an aggregate 88 complications were recorded; 6 (2.4%) donors experienced multiple complications. 3 (1.2%) donors had intraoperative complications. Short-term (≤3 months) complications in 70/82 (85.4%) donors, long-term (>3 months) complications in 12/82 (14.6%) donors. Mortality There was no donor mortality. Follow-up Mean of 9.3 years (range=0.5–14.1)
Li, 2012^17^	China	129 (108 vs 21)	Range=19–65 years	Close relatives	RL (129)	2005–2009	Complications <50=31/108 (28.7%) <50 vs ≥50; *P*=0.719 ≥50=8/21 (38.1%) Mortality No donor death. Follow-up Mean of 3.8 ± 1.8 years.
Muzaale, 2012^12^	United States	4111 (3691 vs 420)	Range=≥17 years	Parent (1035), child (1034), sibling (648), other biological (420), other nonbiological (713), spouse or partner (249), missing (12)	RL (2742), LL (359), LLS (996), missing (14)	Apr. 1994–Mar. 2011 25 Oct., 1999–Mar. 2011 in analysis	Complications: catastrophic events excluding death <50=3/3691 (<0.1%) ≥50=1/420 (0.2%) one donor experienced sub fulminant liver failure (age 20s), and 3 acute liver failure (age 20s, 40s and 50s). Mortality: early death (≤90 days) <50=5/3691 (0.1%) ≥50=2/420 (0.5%) 7 LLDs (age 20s, 30s, 30s, 30s, 40s, 50s and 50s) died ≤90 days after donation. No obvious correlates by donor age with early death. Follow-up Median of 7.6 years (IQR=4.2–10.1).
Nakamura, 2021^18^	Japan	95 (64 vs 31)	34.5 (SD=9.5) –56.9 (SD=4.4) years	Unclear	RL (57), LL (38)	2003–2017	Complications ≤50=25/64 (39.1%) ≤50 vs >50; *P*=0.82 >50=11/31 (35.5%) Biliary leakage in 12.5% in group Y and in 6.5% in group O (*P*=0.49). Most frequent: wound infection in 12.5% in group Y and in 16.1% in group O. Mortality No surgery-related fatality in donors. Follow-up Median of 8.1 years (range=0.1–17.7).
Shackleton, 2005^24^	United States	37 (32 vs 5)	Range=20–62 years	Related by blood, marriage of life-partner relationship (25), friends (12)	RL (34), LLS (3)	≥1999	Complications <50=7/32 (21.9%) ≥50=1/5 (20%) 8/37 (21.6%) donors exhibited 11 AEs, including 3 grade I (27%), 7 grade II (64%), and 1 grade III events (9%). 6 donors had 1 AE, whereas the others had 2 and 3 AEs. Overall incidence of 0.30 AEs/case. 11 AEs presented clinically in the perioperative period, five (45%) within 1–30 days, and 1 (9%) >1 month. Mortality No deaths occurred. Follow-up Median of 2.8 years (range=0.5–5.1)
Suh, 2015^19^	South Korea	886 (840 vs 46)	Range=20–55 years	3^rd^ degree consanguinity or an intense emotional relationship Relatives (818)	RL (704), LL (57), LLS (88), RPS (3), ERL (34)	Jan. 1999-Dec. 2012	Complications ≤50=129/840 (15.4%) >50=6/46 (13%) Most complications were C-D I (73.3%) and II (24.4%). 7 cases of C-D IIIa complications. Mortality No progression to death. Follow-up Mean of 11.7 ± 1.6 in 1999–2004, 5.7 ± 1.8 in 2005–2010, and 2.1 ± 0.6 years in 2011–2012
Tokodai, 2016^20^	Japan	56 (46 vs 10)	Range=18–64 years	Related (44), unrelated (12)	RL (52), LL (4)	Apr. 2001–Aug. 2010	Complications <50=8/46 (17.4%) ≥50=3/10 (30%) The complication rates did not differ between the Y and O group
Wang, 2015^21^	China	159 (149 vs 10)	Median=26 (IQR=23–36) years	Family relatives	Unclear	Mar. 2007–Dec. 2011 Follow-up until July 2013	Complications <50=8/149 (5.4%) ≥50=1/10 (10%) Mortality No donor died in perioperation. Follow-up Average of 5.1 ± 0.4 (range=1.1–5.5) and 5.5 ± 0.1 years (range=0.1–6.1) in the O and Y groups, respectively
Yeow, 2022^22^	Singapore	90 (79 vs 11)	Mean=25.7 (SD=0.5) –54.6 (SD=0.9) years	Unclear	RL (83), LL (6), LLS (1)	Nov. 1996–Dec. 2019	Complications 20–29=1/27 (3.7%) 30–49=3/52 (5.8%) ≥50=0/11 (0%) Mortality No donor mortality

AE, adverse event; C–D, Clavien–Dindo; ERL, extended right lobe; group O, group older donors; group Y, group younger donors; IQR, inter quartile range; LL, left lobe; LLD, living liver donors; LLS, left lateral segment; RL, right lobe; RPS, right posterior segment.

Consanguinity examples: 1^st^ degree=parents; 2^nd^ degree=brothers/sisters; 3^rd^ degree=nephews/nieces/uncles/aunts; 4^th^ degree=grandnephews/grandnieces.

* *P* <0.05.

#### Results complications

**Figure 2:**
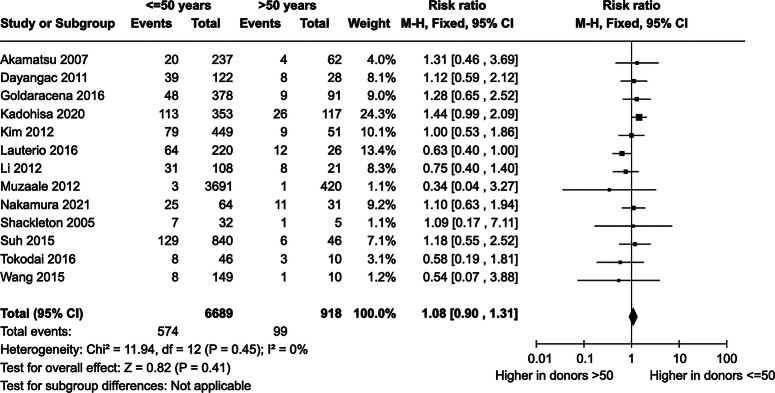
Forest plot for donor complications in younger and older living liver donors.

As shown in Table 1 and Figure 2, donors with complications (%) were discussed in 13 studies^12,14–21,23–26^. Of these studies, Suh *et al*.^19^ had the shortest follow-up with a median of 2.1 years, and Lauterio *et al*.^26^ the longest with a mean of 9.3 years (Table 1). Complication data was recorded for 7607 donors, of which 6689 (87.9%) were younger donors and 918 (12.1%) older donors. In 673 donors, complications occurred, of which 574 (85.4%) in younger donors and 99 (14.6%) in older donors. Overall complication rate was 673/7607=8.8%. Five studies^12,17,20,21,26^ reported a higher risk of complications in younger donors, seven studies^14,18–20,23–25^ in older donors, and one study^16^ reported no difference in complication risk. The risk ratio for complications in younger donors was 1.08 [0.90–1.31] (*P*=0.41). SDC, Fig. 1, Supplemental Digital Content 1, http://links.lww.com/JS9/C320 shows the sensitivity analysis with random effects.

#### Subgroup analysis for major donor complications

Studies reported Clavien–Dindo grades of complications in different ways and/or used different Clavien–Dindo grades as a cut-off for major complications. Dayangac *et al*.^25^, Kadohisa *et al*.^15^, and Li *et al*.^17^ reported both IIIa and IIIb complications, Goldaracena *et al*.^23^, Nakamura *et al*.^18^, and Tokodai *et al*.^20^ IIIb complications ≤30 days, Lauterio *et al*.^26^ III complications, and Muzaale *et al*.^12^ reported catastrophic events. Shackleton *et al*.^24^ did not use the Clavien–Dindo classification but described the complications. We used this to classify the complications according to Clavien–Dindo.

**Figure 3:**
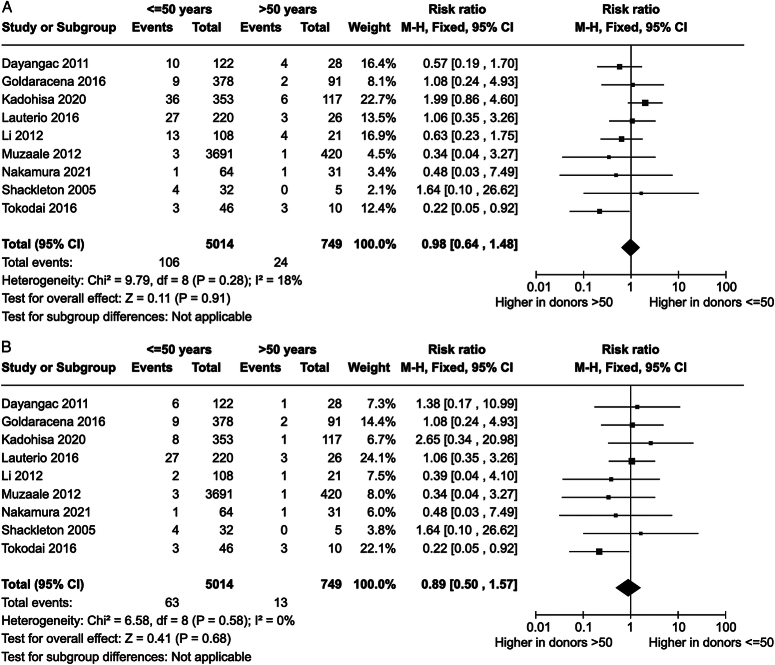
Forest plots for major donor complications in younger and older living liver donors using Clavien–Dindo ≥III (A) and Clavien–Dindo ≥IIIb (B) as a major complication.

When using Clavien–Dindo ≥III as a major complication, the risk ratio for major complications in younger donors was 0.98 [0.64–1.48] (*P*=0.91) (Fig. 3A). When instead using Clavien–Dindo ≥IIIb, the risk ratio for major complications in younger donors was 0.89 [0.50–1.57] (*P*=0.68) (Fig. 3B). SDC, Figs 2 and 3, Supplemental Digital Content 1, http://links.lww.com/JS9/C321 show the sensitivity analysis with random effects.

#### Subgroup analysis for donor biliary complications

**Figure 4:**
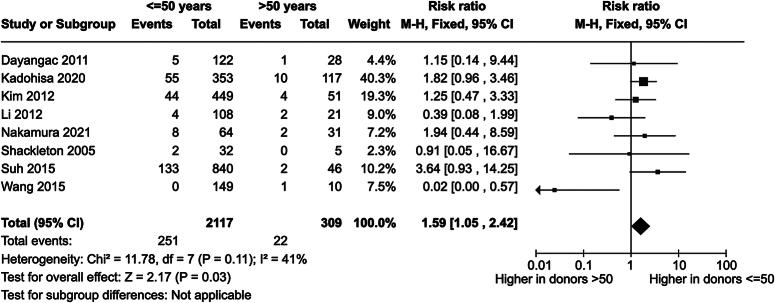
Forest plot for donor biliary complications in younger and older living liver donors.

Seven studies^15–19,21,24^ reported overall donor biliary complications, and one study^25^ major donor biliary complications. The risk ratio for biliary complications in younger donors was 1.59 [1.05–2.42] (*P*=0.03) (Fig. 4). SDC, Fig. 4, Supplemental Digital Content 1, http://links.lww.com/JS9/C321 shows the sensitivity analysis with random effects.

#### Results mortality

As shown in Tables 1, 2, donor mortality was reported in two studies^12,13^. Nine studies^16–19,21,23–26^ discussed donor mortality but reported no events.

**Table 2:** Donor mortality in younger and older living liver donors.

Author, year	Total mortality	Deaths in ≤50 (*n*)	Total ≤50 (*n*)	Deaths in >50 (*n*)	Total >50 (*n*)	Risk ratio [95% CI]
Hong, 2021^13^	53/10116 (0.5%)	42 (0.4%)	9547	11 (1.9%)	569	0.23 [0.12–0.44]
Muzaale, 2012^12^	7/4111 (0.2%)	5 (0.1%)	3691	2 (0.5%)	420	0.28 [0.06–1.46]

n, number.

Mortality data was recorded for 14 227 donors, of which 13 238 (93%) were younger donors and 989 (7%) older donors. A total of 60 deaths occurred, of which 47 (78.3%) in younger donors and 13 (21.7%) in older donors. Overall mortality rate was 60/14 227=0.4%. The mortality rate in younger donors was 47/13 238 (0.4%) and in older donors 13/989 (1.3%).

Muzaale *et al*.^12^ reported seven early deaths (≤90 days) in 4111 donors. Five (0.1%) deaths occurred in younger donors and two (0.5%) in older donors. The risk ratio for mortality in younger donors was 0.28 [0.06–1.46].

Hong *et al*.^13^ reported 53 long-term deaths in 10 116 donors. In younger donors, 42 (0.4%) deaths occurred, and in older donors, 11 (1.9%). The risk ratio for mortality in younger donors was 0.23 [0.12–0.44]. Causes of death were suicide (35.8%), cancer (17%), traffic accident (13.2%), cerebrovascular disease (5.7%), and acute myocardial infarction (1.9%). Five deaths (9.4%) were associated with liver disease, which included one hepatic failure after donor hepatectomy, one acute hepatitis A, one liver cirrhosis, and two alcoholic liver diseases. Nine deaths (17%) were associated with other causes. Univariate analyses found significantly higher mortality among donors aged 50–59 years (HR=6.19; *P*<0.001) or aged ≥60 years (HR=16.49; *P*<0.001) compared to those aged 16–29 years.

#### Quality assessment

The results of the NOS for cohort studies are shown in SDC, Table 1, Supplemental Digital Content 1, http://links.lww.com/JS9/C321. Fourteen studies^12–21,23–26^ had good quality. The study of Yeow *et al*.^22^ had poor quality because there was nothing stated about comparability. Therefore, this study was excluded from the data synthesis. Two good-quality studies^15,20^ did not report the follow-up duration.

#### Funnel plots for complications

SDC, Figure 5, Supplemental Digital Content 1, http://links.lww.com/JS9/C321 shows the funnel plot for the included studies on donor complications. All studies but one were within the funnel. There was a slight asymmetric distribution of studies towards a lower risk ratio. There were no smaller studies with a high risk ratio (right bottom).

SDC, Figure 6, Supplemental Digital Content 1, http://links.lww.com/JS9/C321 shows the funnel plot for the included studies on major donor complications (Clavien–Dindo ≥III). Eight out of nine (89%) studies were within the funnel and there was a lack of small studies with high risk ratios.

SDC, Figure 7, Supplemental Digital Content 1, http://links.lww.com/JS9/C321 shows that for major donor complications (Clavien–Dindo ≥IIIb), there was a symmetric distribution, and all the studies were within the funnel.

SDC, Figure 8, Supplemental Digital Content 1, http://links.lww.com/JS9/C321 shows that for donor biliary complications, 7/8 (88%) studies were within the funnel. There was an asymmetric distribution towards a lower risk ratio and there were no smaller studies with a high risk ratio.

### Quality of life

#### Characteristics of the included studies

The characteristics of the included studies are shown in Table 3. The studies were from India^28^ and Japan^27^. The analyses consisted of 568 donors, of which 414 (72.9%) were younger donors and 154 (27.1%) older donors.

**Table 3:** Characteristics of the two included studies on donor quality of life.

Author, year	Country	Sample size (≤50 vs >50)	Donor age	Donor-recipient type	Surgery technique	Data collection period	Data collection point	Quality of life results
Chandran, 2017^28^	India	200 (182 vs 18)	Range=≥18 years	Adult-adult and adult-pediatric recipient Spouses or relatives (98.5%), unrelated (1.5%)	RL (184), LL (12), LLS (4)	Sep. 2011–June 2014	1 year follow-up	PCS 18–35=52.1 (6.81) 18–35 vs >50; *P*=0.002* 36–50=47.38 (9.98) >50=43.77 (12.02) MCS 18–35=54.41 (4.89) 36–50=52.76 (6.54) >50=52.94 (7.46)
Morooka, 2019^27^	Japan	449, of which 374 in final analysis (232 vs 136; six with unknown age)	Range=20–75 years	Parent (101), child (132), spouse (73), siblings (48), other relatives (16), unknown (4)	Partial hepatectomy	1992–2011	>1 year post donation	PCS 20–29=53.8 (9.4) 30–39=54.1 (6.9) 40-49=53.7 (6.0) 50–59=52.2 (8.6) 60–69=53.0 (4.2) 70–79=49.3 (4.3) MCS 20–29=50.7 (12.1) 20–29 vs 70–79; *P*=0.04* 30–39=52.7 (10.8) 30–39 vs 60–69; *P*=0.04* 40–49=53.1 (10.0) 50–59=54.5 (9.2) 60–69=57.0 (7.5) 70–79=58.7 (4.6)

LL, left lobe; LLS, left lateral segment; MCS, mental component summary; PCS, physical component summary; RL, right lobe.

* *P* <0.05.

## Results

In Chandran *et al*.^28^, mean (SD) PCS was 49.25 (8.73) in younger (*n*=182) and 43.77 (12.02) in older donors (*n*=18); mean difference=5.48 [−0.22–11.18] (Table 4A). In Morooka *et al*.^27^, mean (SD) PCS was 53.92 (6.94) in younger (*n*=232) and 52.28 (6.67) in older donors (*n*=136); mean difference=1.64 [0.21–3.07] (Table 4A). After the accumulation of both studies, mean PCS in younger donors was 51.87 and in older donors 51.29.

**Table 4:** Physical (A) and mental (B) component summary in younger and older living liver donors.

Author, year	Mean (SD) in ≤50	Total ≤50 (n)	Mean (SD) in >50	Total >50 (*n*)	Mean difference [95% CI]
Chandran, 2017^28^	49.25 (8.73)	182	43.77 (12.02)	18	5.48 [−0.22–11.18]
Morooka, 2019^27^	53.92 (6.94)	232	52.28 (6.67)	136	1.64 [0.21–3.07]
Chandran, 2017^28^	53.41 (5.89)	182	52.94 (7.46)	18	0.47 [-3.08–4.02]
Morooka, 2019^27^	52.56 (10.71)	232	55.73 (8.24)	136	−3.17 [-5.12–1.22]

n, number.

In Chandran *et al*.^28^, mean (SD) MCS was 53.41 (5.89) in younger (*n*=182) and 52.94 (7.46) in older donors (*n*=18); mean difference=0.47 [−3.08–4.02] (Table 4B). In Morooka *et al*.^27^, mean (SD) MCS was 52.56 (10.71) in younger (*n*=232) and 55.73 (8.24) in older donors (*n*=136); mean difference=−3.17 [−5.12, −1.22] (Table 4B). After the accumulation of both studies, mean MCS in younger donors was 52.93 and in older donors 55.40.

## Quality assessment

The results of the NOS for cohort studies are shown in SDC, Table 2, Supplemental Digital Content 1, http://links.lww.com/JS9/C321. Both studies^27,28^ had good quality.

## Discussion

This systematic review and meta-analysis including 17 studies^12–21,23–28^ assessed the differences in donor complications, mortality, and QoL between younger and older living liver donors.

There were no significant differences in donor complications between younger and older donors. Common complications included biliary and abdominal complications. There was no significant difference in major donor complications. However, younger donors had a significantly higher chance of biliary complications than older donors.

This higher risk of biliary complications in younger donors is remarkable. Although significant, there were few biliary complications in both groups. Especially in the small older donor group, there were only 22 biliary complications. The study of Suh *et al*.^19^ reported a high deviant risk ratio in younger donors of 3.64. In the study, a possible explanation for this risk ratio is described. In the institution of this study, recently the rate of biliary complications was significantly reduced (1999–2004: 7.9%, 2005–2010: 5%, 2011–2012: 0.9%) because of the introduction of the concept of the exact midplane^19^. In addition, the rate of older donors selected in this institution increased (1999–2004: 2.5%, 2005–2010: 4.7%, 2011–2012: 8.9%)^19^. In summary, the rate of biliary complications decreased, and the rate of older donors increased. This may have contributed to the higher risk ratio in younger donors in this study. The study of Kadohisa *et al*.^15^ had a risk ratio of 1.82. This is remarkable as characteristics, like comorbidities, of older donors were rather worse than better than those of younger donors^15^. With a weight of 40.3%, this study had a significant contribution to the risk ratio for biliary complications. The inclusion of older donors may have been done with more caution because of their age. This may be the reason for the lower risk ratio in older donors.

Muzaale *et al*.^12^ and Hong *et al*.^13^ both showed higher mortality risk ratios for younger donors. However, with the 95% CI of 0.06–1.46 in the study of Muzaale *et al*.^12^, this risk ratio cannot be significant. An important difference between these two studies is that Muzaale *et al*.^12^ reported early death (≤90 days) and Hong *et al*.^13^ long-term deaths. This difference may explain the higher mortality rates in the study of Hong *et al*.^13^. This may also be the reason for the higher risk in older donors. The risk of death of 1.9% in older donors is high. However, for long-term deaths, it is logical that older people had a relatively high mortality risk as this is also the case in the general population. In addition, only 9.4% of deaths were associated with liver disease and can be related to donation. In conclusion, these results need to be interpreted with caution due to the influence of the causes of death.

The high number of suicidal events is remarkable. However, in South Korea, where this study was performed, suicide is the first and/or second cause of death in various age categories from 20 to 59 years old^29^.

When interpreting these donor mortality results, it is important to consider that nine studies^16–19,21,23–26^ reported no deaths.

Morooka *et al*.^27^ reported a better physical QoL in younger donors and a better mental QoL in older donors. Chandran *et al*.^28^ reported a mean difference in PCS of 5.48 [−0.22, 11.18]. However, due to this 95% CI, this cannot be a significant difference. This is also the case for the mean difference in MCS. Furthermore, it seems logical that younger donors may have had a better physical QoL, as that is inherent to being younger. To a certain extent, the reverse applies to mental QoL. Most elderly are mentally stronger because of their life experience^30^. Therefore, it is logical that older donors may have had a higher mental QoL.

Nowadays, the age limit of living liver donors in most transplant centers is 50 years. Therefore, there are very few relevant studies discussing older age cut-offs. Hopefully, this study will contribute to the acceptance of older donors so the optimal age limit can be better determined in the future.

Furthermore, the concept of biological age is gaining significant attention as a potentially superior alternative to chronological age in assessing an individual’s health status^31^. Thus, it may better predict whether someone is eligible for living liver donation. How this biological age relates to chronological age, and what this means for the screening of living liver donors, is an unresolved problem that needs to be studied.

### Limitations

Potentially harmful to this study was nonreporting bias, which occurs when the *P-*value, magnitude or direction of results influences decisions about how, when, or where to report results of eligible studies^32^. The funnel plot for the included studies on donor complications showed that 12/13 (92.3%) studies were within this funnel. There was a lack of small studies with a high risk ratio, which may indicate bias due to missing results. This may indicate an overestimation of the risk ratio^32^. This means that the results may be less in favor of older donors. However, the sensitivity analysis with random effects showed no indication for small-study effects and even when this overestimation would be present, it would be small. With a risk ratio of 1.08, this would not change the results significantly. The same applies to major donor complications (Clavien–Dindo ≥III). However, for donor biliary complications, the asymmetric distribution and possible overestimation may indicate that the risk ratio is actually non-significant. In addition, the sensitivity analysis with random effects resulted in a nonsignificant result. Therefore, this result needs to be interpreted with caution. For major donor complications (Clavien–Dindo ≥IIIb), nonreporting bias is not suspected.

Another potentially harmful limitation was selection. The selection criteria for living liver donors are commonly very strict. Only proven very healthy individuals qualified. Because of their above-average health, they may have been less prone to complications and mortality after surgery. In addition, their QoL before and after LDLT may have also been above average. These effects may have been higher for older donors than for younger donors. LDLT is less commonly performed with older donors and therefore, healthcare professionals were extra careful when selecting these older donors. Only the healthiest older potential living liver donors were selected. As a result, our study may have presented more beneficial outcomes for older donors. However, due to the highly nonsignificant risk ratios for donor complications and major donor complications, this bias is not expected to change the results significantly. For donor biliary complications, it may result in a nonsignificant risk ratio.

While two good-quality studies^15,20^ did not report the follow-up duration, the overall complication rates may have been underestimated. This is not expected to change other results as this influenced both younger and older donors.

Furthermore, the small number of two included studies for both donor mortality and QoL was a limitation. Beforehand, we chose three studies as a minimum to conduct a meta-analysis. Therefore, no meta-analyses were conducted for these outcomes, which made it harder to draw conclusions.

## Conclusion

In conclusion, older donors do not have a higher complication rate or mortality rate than younger donors after LDLT. Neither do they have a higher rate of major complications. Older donors may have a lower rate of biliary complications than younger donors. To make a clear statement about donor mortality, more studies and a meta-analysis on early mortality are needed. In addition, older donors have a similar QoL after LDLT compared to younger donors. However, more studies are needed to conduct a meta-analysis on this subject. With careful selection, older donors can be included in screening programs for living liver donation to expand the donor pool.

## Ethical approval

Not relevant as this study did not involve patients.

## Consent

Not relevant as this study did not involve patients.

## Sources of funding

This research did not receive any specific grant from funding agencies in the public, commercial, or not-for-profit sectors.

## Author contribution

H.W.t.B.: was the principal investigator and was responsible for the study concept, data collection, data analysis and interpretation, and writing the paper; A.J.C.: helped with the data collection and analysis; W.G.P. and L.W.K.: helped with the study concept; M.U.B.: helped with the study concept, data analysis and interpretation, and writing the paper; R.C.M.: was the corresponding author and was also responsible for the study concept, data collection, data analysis and interpretation, and writing the paper.

## Conflicts of interest disclosure

The authors declare that they have no conflicts of interest.

## Research registration unique identifying number (UIN)

PROSPERO: CRD42022322601 https://www.crd.york.ac.uk/prospero/display_record.php?RecordID=322601.

## Guarantor

Dr. Robert C. Minnee, Department of Hepato-Pancreato-Biliary/Transplant Surgery. E-mail: r.minnee@erasmusmc.nl.

## Data availability statement

I confirm that any datasets generated during and/or analyzed during the current study are publicly available, available upon reasonable request.

## Provenance and peer review

Not commissioned, externally peer-reviewed.

## Presentation

This study was presented on the 2023 Joint International Congress of ILTS, ELITA and LICAGE, the 2023 Bootcongres, and the 2023 ESOT Congress.

## Supplementary Material

**Figure:**