# Supplementary material for: Older living liver donors can enlarge the donor pool: a systematic review and meta-analysis
Source: Int J Surg. 2024 Apr 3;110(8):5022–33. doi: 10.1097/JS9.0000000000001419 (PMC11325888; doi:10.1097/JS9.0000000000001419)
Supplement: Supplementary file 1 [file js9-110-5022-s001.pdf]

Older Living Liver Donors can enlarge the Donor Pool: A Systematic Review and Meta-Analysis

SUPPLEMENT DIGITAL CONTENT

SDC, Table 1. Quality assessment of the 15 included studies on donor complications and mortality.

Author, year	Selection				Comparability		Outcome			Methodological quality
	1	2	3	4			1	2	3	
**Akamatsu, 2007**	*	*	*	*	*	*	*	*	*	Good
**Dayangac, 2011**	*	*	*	*	*	*	*	*	*	Good
**Goldaracena, 2016**	*	*	*	*	*	*	*	*	*	Good
**Hong, 2021**	*	*	*	*	*	*	*	*	*	Good
**Kadohisa, 2020**	*	*	*	*	*	*	*		*	Good
**Kim, 2012**	*	*	*	*	*	*	*	*	*	Good
**Lauterio, 2016**	*	*	*	*	*	*	*	*	*	Good
**Li, 2012**	*	*	*	*	*	*	*	*	*	Good
**Muzaale, 2012**	*		*	*	*	*	*	*	*	Good
**Nakamura, 2021**	*	*	*		*		*	*	*	Good
**Shackleton, 2005**	*		*	*	*	*	*	*	*	Good
**Suh, 2015**	*	*	*	*	*	*	*	*	*	Good
**Tokodai, 2016**	*	*	*		*		*		*	Good
**Wang, 2015**	*	*	*	*	*	*	*	*	*	Good
**Yeow, 2022**	*	*	*	*			*		*	Poor

SDC, Table 2. Quality assessment of the 2 included studies on donor quality of life.

Author, year	Selection				Comparability		Outcome			Methodological quality
	1	2	3	4			1	2	3	
**Chandran, 2017**	*		*	*	*			*	*	Good
**Morooka, 2019**	*	*	*	*	*	*		*	*	Good

Study or Subgroup	<=50 years Events	<=50 years Total	>50 years Events	>50 years Total	Weight	Risk ratio M-H, Random, 95% CI
Akamatsu 2007	20	237	4	62	3.2%	1.31 [0.46 , 3.69]
Dayangac 2011	39	122	8	28	8.4%	1.12 [0.59 , 2.12]
Goldaracena 2016	48	378	9	91	7.6%	1.28 [0.65 , 2.52]
Kadohisa 2020	113	353	26	117	25.0%	1.44 [0.99 , 2.09]
Kim 2012	79	449	9	51	8.8%	1.00 [0.53 , 1.86]
Lauterio 2016	64	220	12	26	16.1%	0.63 [0.40 , 1.00]
Li 2012	31	108	8	21	8.9%	0.75 [0.40 , 1.40]
Muzaale 2012	3	3691	1	420	0.7%	0.34 [0.04 , 3.27]
Nakamura 2021	25	64	11	31	10.8%	1.10 [0.63 , 1.94]
Shackleton 2005	7	32	1	5	1.0%	1.09 [0.17 , 7.11]
Suh 2015	129	840	6	46	5.9%	1.18 [0.55 , 2.52]
Tokodai 2016	8	46	3	10	2.7%	0.58 [0.19 , 1.81]
Wang 2015	8	149	1	10	0.9%	0.54 [0.07 , 3.88]
**Total (95% CI)**		**6689**		**918**	**100.0%**	**1.02 [0.85 , 1.23]**
Total events:	574		99			

Heterogeneity: Tau² = 0.00; Chi² = 11.94, df = 12 (P = 0.45); I² = 0%
Test for overall effect: Z = 0.24 (P = 0.81)
Test for subgroup differences: Not applicable

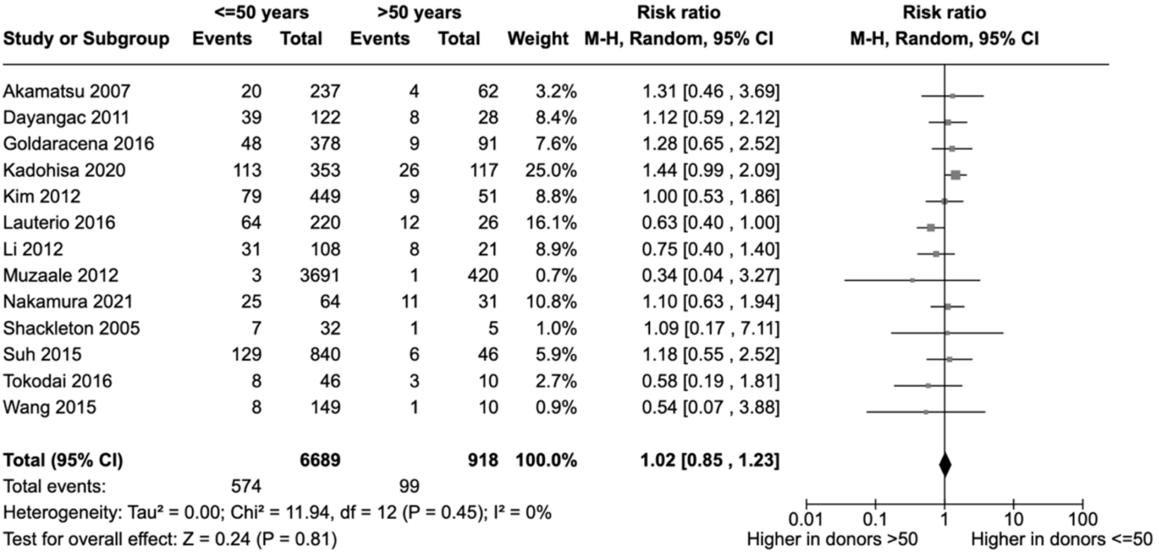

SDC, Figure 1. Forest plot for donor complications in younger and older living liver donors using random effects.

Study or Subgroup	<=50 years Events	<=50 years Total	>50 years Events	>50 years Total	Weight	Risk ratio M-H, Random, 95% CI
Dayangac 2011	10	122	4	28	15.7%	0.57 [0.19 , 1.70]
Goldaracena 2016	9	378	2	91	9.2%	1.08 [0.24 , 4.93]
Kadohisa 2020	36	353	6	117	22.5%	1.99 [0.86 , 4.60]
Lauterio 2016	27	220	3	26	14.9%	1.06 [0.35 , 3.26]
Li 2012	13	108	4	21	17.2%	0.63 [0.23 , 1.75]
Muzaale 2012	3	3691	1	420	4.5%	0.34 [0.04 , 3.27]
Nakamura 2021	1	64	1	31	3.1%	0.48 [0.03 , 7.49]
Shackleton 2005	4	32	0	5	3.0%	1.64 [0.10 , 26.62]
Tokodai 2016	3	46	3	10	9.9%	0.22 [0.05 , 0.92]
**Total (95% CI)**		**5014**		**749**	**100.0%**	**0.82 [0.50 , 1.34]**
Total events:	106		24			

Heterogeneity: Tau² = 0.10; Chi² = 9.79, df = 8 (P = 0.28); I² = 18%
Test for overall effect: Z = 0.80 (P = 0.42)
Test for subgroup differences: Not applicable

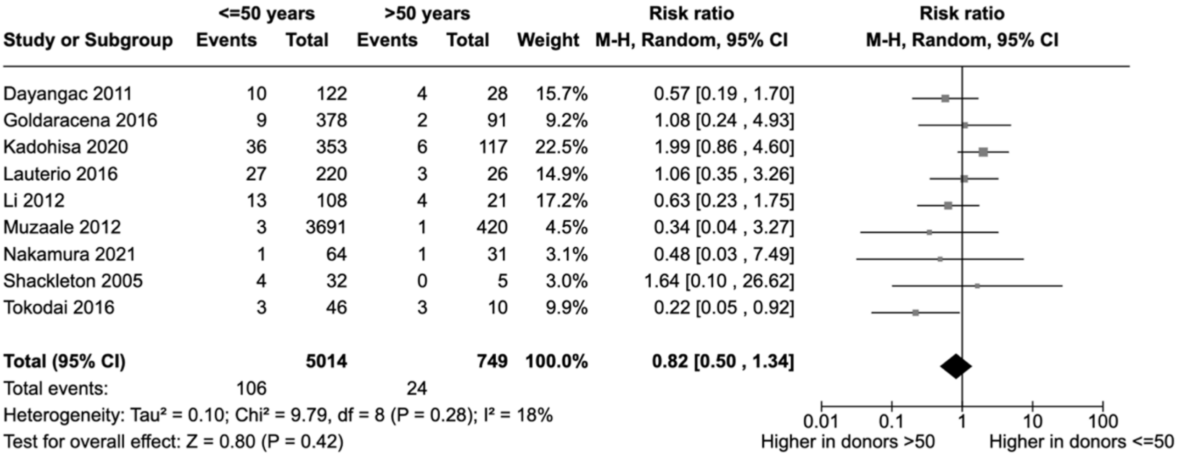

SDC, Figure 2. Forest plot for major donor complications (Clavien-Dindo ≥III) in younger and older living liver donors using random effects.

	<=50 years		>50 years			Risk ratio
Study or Subgroup	Events	Total	Events	Total	Weight	M-H, Random, 95% CI
Dayangac 2011	6	122	1	28	8.2%	1.38 [0.17 , 10.99]
Goldaracena 2016	9	378	2	91	15.5%	1.08 [0.24 , 4.93]
Kadohisa 2020	8	353	1	117	8.3%	2.65 [0.34 , 20.98]
Lauterio 2016	27	220	3	26	28.3%	1.06 [0.35 , 3.26]
Li 2012	2	108	1	21	6.4%	0.39 [0.04 , 4.10]
Muzaale 2012	3	3691	1	420	7.0%	0.34 [0.04 , 3.27]
Nakamura 2021	1	64	1	31	4.7%	0.48 [0.03 , 7.49]
Shackleton 2005	4	32	0	5	4.6%	1.64 [0.10 , 26.62]
Tokodai 2016	3	46	3	10	17.0%	0.22 [0.05 , 0.92]
**Total (95% CI)**		**5014**		**749**	**100.0%**	**0.76 [0.42 , 1.39]**
Total events:	63		13			

Heterogeneity: Tau² = 0.00; Chi² = 6.58, df = 8 (P = 0.58); I² = 0%
Test for overall effect: Z = 0.88 (P = 0.38)
Test for subgroup differences: Not applicable

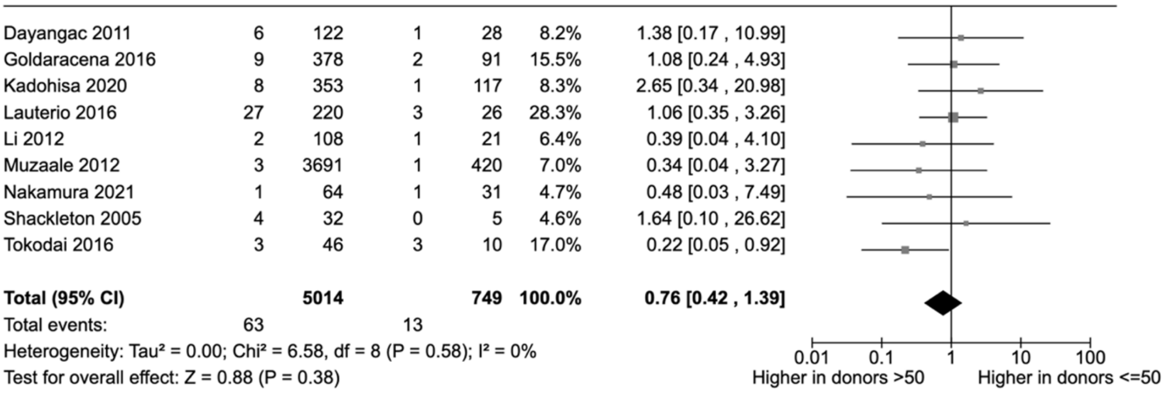

SDC, Figure 3. Forest plot for major donor complications (Clavien-Dindo ≥IIIb) in younger and older living liver donors using random effects.

	<=50 years		>50 years			Risk ratio
Study or Subgroup	Events	Total	Events	Total	Weight	M-H, Random, 95% CI
Dayangac 2011	5	122	1	28	7.7%	1.15 [0.14 , 9.44]
Kadohisa 2020	55	353	10	117	26.3%	1.82 [0.96 , 3.46]
Kim 2012	44	449	4	51	19.7%	1.25 [0.47 , 3.33]
Li 2012	4	108	2	21	11.2%	0.39 [0.08 , 1.99]
Nakamura 2021	8	64	2	31	12.6%	1.94 [0.44 , 8.59]
Shackleton 2005	2	32	0	5	4.5%	0.91 [0.05 , 16.67]
Suh 2015	133	840	2	46	14.0%	3.64 [0.93 , 14.25]
Wang 2015	0	149	1	10	3.9%	0.02 [0.00 , 0.57]
**Total (95% CI)**		**2117**		**309**	**100.0%**	**1.25 [0.64 , 2.42]**
Total events:	251		22			

Heterogeneity: Tau² = 0.33; Chi² = 11.78, df = 7 (P = 0.11); I² = 41%
Test for overall effect: Z = 0.66 (P = 0.51)
Test for subgroup differences: Not applicable

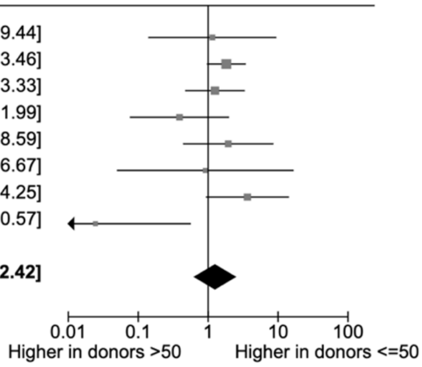

SDC, Figure 4. Forest plot for donor biliary complications in younger and older living liver donors using random effects.

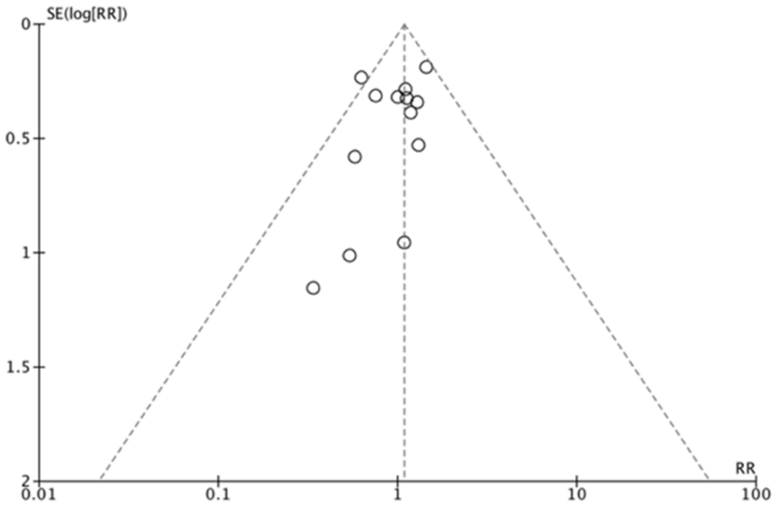

SDC, Figure 5. Funnel plot for the included studies on donor complications in younger and older living liver donors.

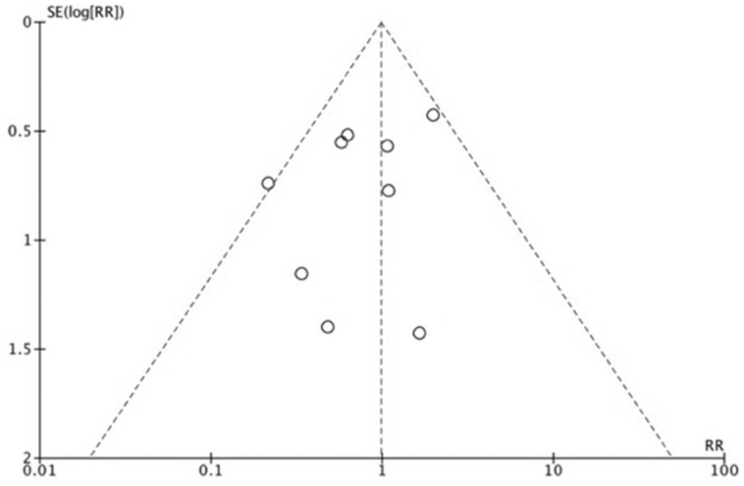

SDC, Figure 6. Funnel plot for the included studies on major donor complications (Clavien-Dindo ≥III) in younger and older living liver donors.

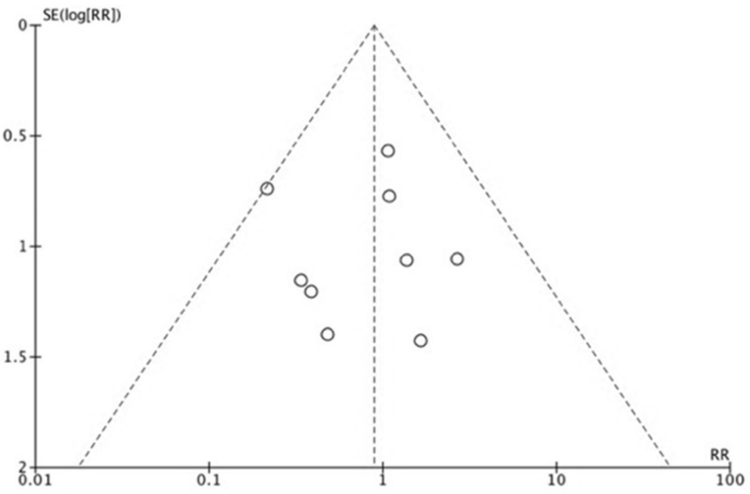

SDC, Figure 7. Funnel plot for the included studies on major donor complications (Clavien-Dindo ≥IIIb) in younger and older living liver donors.

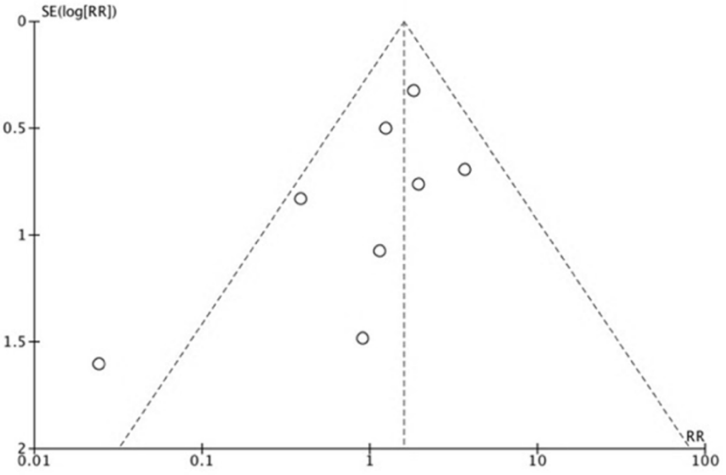

SDC, Figure 8. Funnel plot for the included studies on donor biliary complications in younger and older living liver donors.

APPENDIX 1

FULL SEARCH STRATEGY

Embase

('living donor liver transplantation'/de OR 'living donor liver transplant'/de OR ('living donor'/de AND ('liver transplantation'/exp OR 'liver donor'/de)) OR (((living-donor* OR live-donor*) NEAR/3 (liver- transplant*)) OR LDLT OR living-liver-donat*):ab,ti) **AND** ('mortality'/de OR 'survival'/exp OR 'complication'/exp OR 'complication':lnk OR 'small for size syndrome'/de OR 'reoperation'/de OR 'quality of life'/exp OR (qualit*-of-life* OR QoL OR hrQoL* OR mortalit* OR survival* OR complication* OR small-for-size-syndrome* OR reoperat* OR re-operat*):ab,ti) NOT ((animal/exp OR animal*:de OR nonhuman/de) NOT ('human'/exp)) NOT ('review'/exp OR 'case report'/de OR (review* OR SR OR meta-analys* OR case-report*):ti OR [conference abstract]/lim OR [letter]/lim OR [review]/lim) AND [english]/lim AND [2002-2030]/py

MEDLINE (Ovid)

((Living Donors/ AND Liver Transplantation/) OR (((living-donor* OR live-donor*) ADJ3 (liver- transplant*)) OR LDLT OR living-liver-donat*).ab,ti.) **AND** (Mortality/ OR mortality.fx. OR Survival/ OR Quality of Life/ OR complications.fx. OR Reoperation/ OR (qualit*-of-life* OR QoL OR hrQoL* OR mortalit* OR survival* OR complication* OR small-for-size-syndrome* OR reoperat* OR re- operat*).ab,ti.) NOT (exp animals/ NOT humans/) NOT ((review* OR SR OR meta-analys* OR case- report*).ti.) NOT (news OR congres* OR abstract* OR book* OR chapter* OR dissertation abstract* OR review* OR case report* OR letter*).pt. AND english.lg. AND 2002:2030.(sa_year)

Cochrane Central

((((living NEXT donor* OR live NEXT donor*) NEAR/3 (liver NEXT transplant*)) OR LDLT OR living NEXT liver NEXT donat*):ab,ti) **AND** ((qualit* NEXT of NEXT life* OR QoL OR hrQoL* OR mortalit* OR survival* OR complication* OR small NEXT for NEXT size NEXT syndrome* OR reoperat* OR re NEXT operat*):ab,ti) NOT "conference abstract":pt
